# 

**Published:** 1836-07-01

**Authors:** 


### THE MEDICO-CHIRURGICAL REVIEW, AND JOURNAL OF PRACTICAL MEDICINE.

*(NEW SERIES.)*

VOLUME TWENTY-FIVE,

[1st of APRIL to 30th of SEPTEMBER,]

1836.

VOL. V. of DECENNIAL SERIES.

EDITED

BY JAMES JOHNSON, M.D.

PHYSICIAN EXTRAORDINARY TO THE KING,

AND

HENRY JAMES JOHNSON, ESQ.

LECTURER ON ANATOMY AT THE SCHOOL OF ST. GEORGE'S HOSPITAL IN KINNERTON STREET.

57465
Surgeon General's Office LIBRARY Washington, D.C.

LONDON:

PUBLISHED BY S. HIGHLEY, 32, FLEET STREET,

*Re-printed in New York, by Mr. Wood.*

1836.

WI
ME 75 T

PRINTED BY F. HAYDEN,
Little College Street, Westminster.

### BIBLIOGRAPHICAL RECORD;

OR,

*Works received for Review since last Quarter.*

1. A Treatise on the Chemical, Medicinal, and Physiological Properties of Creosote, illustrated by Experiments on the Lower Animals, &c. being the Harveian Prize-Dissertation for 1836. By John Rose Cormack, Member of the Royal Medical and Physical Societies of Edinburgh. 1836.

---

2. Guy's Hospital Reports. No. 2, April, 1836. Edited by George H. Barlow, M.A. and James P. Babington, M.A. Price Four Shillings. Highley, 1836.

---

3. Quackery: its Danger, Irrationality, and Injustice; the Causes of its Success, the best Means for its Suppression. Addressed to all Classes. Bath, April, 1836, price Sixpence.

---

4. Outlines of Human Pathology. By Herbert Mayo, F.R.S. &c. Part II. 1836.

---

5. Factory Statistics. The Official Tables appended to the Report of the Select Committee on the Ten-Hour Factory Bill, &c. By the late T. M. Sadler, Esq. Octavo, pp. 80. London, 1836.

---

6. A Letter to Lord John Russell, on the Condition of the Pauper Children of St. James's, Westminster. By T. J. Pettigrew, F.R.S. Octavo, pp. 43. April, 1836.

---

7. The Dublin Journal of Medical Science, &c. May, 1836. *In Exchange.*

---

8. Medical Commentaries on Puerperal Fever, Vermination, and Water in the Head. By John Alexander, M.D. Octavo, pp. 69. 1835.

---

9. Isis Revelata—an Inquiry into the Origin, Progress, and present State of Animal Magnetism. By J. C. Colquhoun, Esq. Advocate, F.R.S. &c. Two Volumes, 8vo, pp. 700. Maclachlan and Stewart, Edinb. April, 1836.

10. Lectures on Subjects connected with Clinical Medicine. By P. M. Latham, M.D. Physician to St. Bartholomew's Hospital. Octavo, pp. 322. Longman and Co. April, 1836.

---

11. The Physiology of Digestion, considered with regard to the Principles of Dietetics. By Andrew Combe, M.D. Octavo, pp. 332. Maclachlan and Co. April, 1836.

☞ *This may be considered as a continuation of the author's very popular work, "the Principles of Physiology, applied to the Preservation of Health," and is executed with the same talent and learning displayed in that publication.*

---

12. An Introductory Address, delivered at Apothecaries' Hall, to the Members of that Society, 1835. By Henry Field. Ocavo, sewed. 1836.

☞ *A very appropriate and well-written address.*

---

13. The Cyclopædia of Anatomy and Physiology. Edited by Robert B. Todd, M.D. Part VI., containing "*Cavity*," by Dr. Todd—"*Cellular Tissue*," by R. D. Grainger, Esq.—"*Cephalopoda*," by R. Owen, Esq.—"*Cerumen*," by W. T. Brande, Esq.—"*Cetacea*," by Mons. F. Cuvier—"*Cheiroptera*," by T. Bell, Esq. —"*Chyliferous System*," by Dr. Grant— "*Cicatrix*," by A. T. S. Dodd, Esq.— "*Celia*," by Dr. Sherpy.

☞ *This is a highly respectable part of an excellent and praiseworthy undertaking.*

---

14. The Clinique Médicale; or Report of Medical Cases. By G. Andral. Considered and translated, with Observations extracted from the Writings of the most distinguished Medical Authors, by D. Spillan, M.D. &c. Part IV. containing Diseases of the Abdomen. Renshaw, Strand, May, 1836. Price Five Shillings.

☞ *This is a very valuable and interesting part of a highly important Work.*

15. Pathological Observations on the Diseases of the Placenta. Part I. Congestion and Inflammation. By JAMES Y. SIMPSON, M.D. Read before the Royal Medical Society of Edinburgh. Nov. 20th, 1835.

☞ *This valuable paper is published in a recent Number of the Edinburgh Medical and Surgical Journal, and is highly deserving of the attention of pathologists in general, and accoucheurs in particular.*

---

16. St. Thomas's Hospital Reports. By JOHN F. SOUTH. No. 3. April, 1836.

---

17. A Practical Essay on the History and Treatment of Beriberi. By Assistant-Surgeon JOHN GRANT MALCOLMSON, of the Madras Establishment. Octavo, pp. 399. Madras, 1835.

---

18. Lectures on the Nervous System and its Diseases. By MARSHALL HALL, M D. &c. Pp. 170. May, 1836.

---

19. Authentic Report of an Introductory Lecture on the important Subject of Medical Reform, delivered in the School of Anatomy, Medicine, and Surgery, Peter-street, Dublin. By ANDREW ELLIS, Esq. Octavo, pp. 63. Dublin, 1836.

---

20. Observations on the Medical and Surgical Agency of the Air-Pump. Read before the British Association in 1835. By Sir JAMES MURRAY, M.D. Octavo, pp. 63. Dublin, 1836.

---

21. *Library of the Medical Sciences.*—The American Cyclopædia of Practical Medicine and Surgery; a Digest of Medical Literature. Edited by ISAAC HAYS, M.D. Parts 8 and 9. Philadelphia, 1835.

☞ *This important Work has only yet reached the article* "ARTERIES." *It bids fair to diffuse medical science through the United States, to an extent hitherto unexampled.*

---

22. On the Antidotal Treatment of Cholera; with a Sketch of the Physiology of the Disease, as deduced from that of intermittent Fever. By J. PARKIN, M.R.C.S. &c. Octavo, pp. 112. Highley, 1836.

---

23. Outlines of Comparative Anatomy. By ROBERT GRANT, M.D. Professor of Comparative Anatomy and Zoology in the University of London, &c. Part the Third, containing the Nervous System, Organs of Sense, and Digestive Organs, with 23 Wood-cuts. Octavo, price 5s. Baillière, London, May, 1836.

---

24. An Inquiry into the Pathology, Causes, and Treatment of Puerperal Fever; being an Essay for which the Fothergillian Gold Medal was conferred on the Author by the Medical Society of London, in March, 1835. Octavo, pp. 247. Highley, 1836.

---

25. A Sketch of the Medical Monopolies, with a Plan of Reform. Addressed to the Right Hon. Lord John Russell. By JAMES KENNEDY, M.R.C.S. L. Author of the History of Cholera. Octavo, pp. 95. Highley, 1836.

---

26. On Phlebitis, particularly as connected with Secondary Abscesses:—An Essay submitted to the Faculty of Physicians and Surgeons of Glasgow, when Candidate for Admission into that Body. By JAMES DOUGLAS, A.M. &c. late House Surgeon to the Glasgow Royal Infirmary. Octavo, pp. 48. 1835.

☞ *We shall introduce some interesting cases from this able thesis in our next Periscope.*

---

27. Address of Earl Stanhope, President of the Medico-Botanical Society, for the Anniversary Meeting, January 16th, 1836.

☞ *This Address, like the noble President himself, is distinguished by suavity, elegance, learning, and science—rare combinations, and reflecting no small lustre on the* ORDER *of aristocracy to which the President of this rising Society belongs!*

---

28. Révue Medico-Chirurgicale Anglaise, et des Sciences Accessoires, &c. Par BUREAUD RIOFREY, M.D. No. I. May 15th, 1836, pp. 48 This new namesake of our's is designed to give our Continental Brethren a fuller account of English theory and practice, than has hitherto been given through the medium of the Continental Journals, in which our medical literature is very little noticed.

---

29. A popular View of Homœopathy, exhibiting the present State of the Science, &c. By the Rev. THOMAS R. EVEREST, Rector of Wickwar. *Second Edit.* 1836.

---

30. Transactions of the Provincial Medical and Surgical Association. Vol. IV. 1836.

---

*Closed on the* 17*th June.*

